# Nutrigenomic profiling of reduced specification diets and phytogenic inclusion effects on critical toll-like receptor signaling, mitogen-activated protein kinase-apoptosis, and PI3K-Akt-mTOR gene components along the broiler gut

**DOI:** 10.1016/j.psj.2023.102675

**Published:** 2023-03-29

**Authors:** Eirini Griela, Konstantinos C. Mountzouris

**Affiliations:** Laboratory of Nutritional Physiology and Feeding, Department of Animal Science, School of Animal Biosciences, Agricultural University of Athens, 11855 Athens, Greece

**Keywords:** diet type, phytogenic, inflammation, apoptosis, metabolism

## Abstract

The effects of concurrent reduction of dietary metabolizable energy (**ME**) and crude protein (**CP**) levels combined or not with the dietary inclusion of a phytogenic feed additive (**PFA**) were studied using a nutrigenomics approach. In particular, the expression of 26 critical genes relevant for inflammation control (*TLR* pathway), cellular apoptosis (*MAPK* pathway) cell growth and nutrient metabolism (*PI3K-Akt-mTOR* pathway) was profiled along the broiler intestine. Two dietary types (L and H) differing in metabolizable energy and crude protein levels (L: 95% and H: 100% of optimal Cobb 500 recommendations for ME and CP requirements) supplemented or not with PFA (− or +) and their interactions (L−, L+, H−, H+) were evaluated. There were only 3 total interactions (*mTOR, IL8*, and *HRAS P* < 0.05) between diet type and PFA inclusion indicating limited concurrent effects. Diet type, L upregulated genes related with inflammation mainly in the jejunum, ileum, and cecum (*P* < 0.05) and *MAPK* pathway in the ileum and cecum (*P* < 0.05). Moreover, diet type L negatively affected the expression of genes related to PI3K-Akt-mTOR pathway mainly in duodenum and cecum (*P* < 0.05). On the other hand, PFA inclusion downregulated (*P* < 0.05) genes related with *TLR* signaling pathway (*TLR2B, MyD88, TLR3, IL8, LITAF*) along the intestine and *MAPK* pathway genes (*APO1, FOS*) in jejunum (*P* < 0.05). Finally, PFA supplementation regulated nutrient sensing and metabolism in the cecum in a manner perceived as beneficial for growth. In conclusion, the study results highlight that the reduced ME and CP specifications, especially in the absence of PFA, regulate inflammation, apoptosis and nutrient metabolism processes at homeostatic control levels that hinder maximizing the availability of dietary energy and nutrients for growth purposes. Inclusion of PFA helped to adjust the respective homeostatic responses and control to levels supporting broiler performance, especially at reduced specification diets.

## INTRODUCTION

Gut health biomarkers are highly warranted at present (Ducatelle et al., 2018). As a result, nutrigenomic studies assessing critical genes related with broiler gut function and health are receiving current scientific attention (Mountzouris et al., 2020; Griela et al., 2021). Genes of certain pathways underpin processes that lead to inflammation, apoptosis, nutrient sensing and protein synthesis in the gut. In particular, the toll-like receptors (***TLRs***) signaling pathway is known to be essential for gut homeostasis and inflammation control due to the ability of TLRs to trigger the activation of nuclear factor (***NFkB1***) and the subsequent production of proteins related to inflammatory response (Kawai and Akira, 2007; Kogut et al., 2012; Kawasaki and Kawai, 2014; Liu et al., 2017; El-Zayat et al., 2019). In addition, the mitogen-activated protein kinase (***MAPK***) pathway, an integral pathway of cell apoptosis, includes genes that have been reported to cause functional damages upon overexpression that affect negatively nutrient digestion-utilization and broiler performance (Yamada et al., 2017; Yue and López, 2020; Zhu et al., 2021). Moreover, the mechanistic target of rapamycin kinase (***mTOR***) combined with the activation of *Akt* in the *PI3K/Akt/mTOR* pathway enhance nutrient sensing, mRNA translation, protein biosynthesis including skeletal muscle cell development (Sarbassov et al., 2005; Zhang et al., 2009; Bottje et al., 2014).

In broiler nutrition, energy and protein dietary levels are critical factors for bird health status and performance efficiency (Neto et al., 2000; Paraskeuas et al., 2016; Taleb et al., 2017; Kidd et al., 2021). The rational reduction of dietary energy and protein levels could be considered as part of overall management strategies targeting feed cost reduction and better overall nutritional efficiency. However, hard data on broiler gut function and health indices that could critically support performance responses are currently limited (Bravo et al., 2014; Shabani et al., 2015; Paraskeuas et al., 2016; Griela et al., 2021). In addition, certain feed additives such as phytogenics (**PFA**) are purported to benefit performance via enhanced nutrient digestibility—metabolism, improved gut microbiota and overall gut health (Mountzouris et al., 2011; Murugesan et al., 2015; Du et al., 2016; Akyurek and Yel, 2019) but their beneficial use in conjunction with reduced dietary specs are still far from clear.

The aim of this present research was to generate new knowledge on the effects of reduced dietary energy and protein levels combined or not with PFA supplementation on certain biomarkers involved in pathways related with inflammation control, cellular apoptosis, nutrient sensing and protein synthesis in the broiler gut. In particular, the expression of critical genes involved in the *TLR* signaling pathway as well as cellular apoptosis (*MAPK* pathway) and cellular growth and metabolism (*PI3K-Akt-mTOR* pathway) were determined using nutrigenomics analysis along the broiler intestine.

## MATERIALS AND METHODS

### Animals, Housing, and Experimental Treatments

This study forms part of our previous research work (Griela et al., 2021) and in order to avoid replication, a short description of the experiment and the treatments is given below. For the experiment 540 one-day-old, male Cobb 500 broilers vaccinated at hatch for Marek, Infectious Bronchitis and Newcastle Disease were used. Birds were allocated to 4 experimental treatments for 6 wk. Each treatment had 9 floor replicate cages, each having 15 broilers each.

The experiment had a 2 × 2 factorial design with diet specifications (i.e., **L, H**) and PFA addition (i.e. **−, +**) as the main factors. The nutritional program was a 3-phase feeding scheme with starter (1–11 d), grower (12–22 d), and finisher (23–42 d) diets meeting optimal Cobb 500 broiler requirements. For each growth period 2 diet types (L and H) were formulated. Diet L was designed to meet 95% and H to meet 100% of the recommended Cobb 500 metabolizable energy (**ME**) and crude protein (**CP**) requirements. The PFA used is a blend of volatile components consisting of encapsulated carvacrol, thymol, D-carvone, methyl salicylate, and L-menthol (13%) and extracts and plant powders from spices and medicinal plants (5%) (Digestarom, Biomin Phytogenics GmbH, Stadtoldendorf, Germany).

Depending on diet type (L and H) and PFA supplementation (0 and 150 mg/kg of diet) the 4 experimental treatments were **L−** (95% of optimal broiler nutrient requirements with no PFA supplementation), **L+** (95% of optimal broiler nutrient requirements with PFA supplementation), **H−** (100% of optimal broiler nutrient requirements with no PFA supplementation) and **H+** (100% of optimal broiler nutrient requirements with PFA supplementation). Birds were euthanized via electrical stunning prior to slaughter. The experimental protocol was in accordance with the current European Union Directive on the protection of animals used for scientific purposes (EC 43/2007; EU 63/2010) and was approved by the relevant national authority (Department of Agriculture and Veterinary Policy, General Directorate of Agriculture, Economy, Veterinary and Fisheries: approval 1130/290216).

At 42 d of age, 9 broilers per treatment (i.e., 1 broiler per replicate cage) were randomly selected, and after euthanasia the duodenum, jejunum, ileum and ceca samples were excised aseptically, snap frozen in liquid nitrogen and stored at −80°C until further analyses.

### Organ Sampling and Molecular Analyses

After euthanasia the duodenum, jejunum, ileum, and ceca samples were excised aseptically, snap frozen in liquid nitrogen and stored at −80°C.

### RNA Isolation and Reverse-Transcription PCR

The middle section (e.g., 15 cm) of duodenum, jejunum, ileum, and the whole ceca were thawed on ice and opened longitudinal and the luminal digesta were removed. Eventually, the sections without digesta were washed efficiently in 30 mL ice cold PBS-EDTA (10 mM) solution (pH = 7.2) and each mucosal epithelium was scraped off with a micro slide and stored in sterile Eppendorf type tube. Ultimately, the extraction of the total RNA from the duodenal, jejunal, ileal, and cecal mucosa was generated by NucleoZOL Reagent (Macherey-Nagel GmbH & Co. KG, Düren, Germany), according to the manufacturer's instructions. Spectrophotometry (NanoDrop-1000, Thermo Fisher Scientific, Waltham, United Kingdom) was used to determine the RNA quantity and quality.

Afterward, for removing potential genomic DNA leftovers from the RNA samples DNAse treatment was applied. Ten micrograms of total RNA were resuspended with 1 U of DNase I (M0303, New England Biolabs Inc., Ipswich, UK) and 10 μL of 10× DNAse buffer for a final volume of 100 µL with the inclusion of DEPC water. For DNAse activation the samples were incubated for 20 min at 37°C. Next, there was an EDTA addition to a final concentration of 5 mM to protect RNA from being degraded and for the DNAse inactivation samples incubated at 75°C for 10 min. RNA integrity was checked by agarose gel electrophoresis.

For cDNA construction, 500 ng of total RNA from each sample were converted to cDNA by PrimeScript RT Reagent Kit (Perfect Real Time, Takara Bio Inc., Shiga-Ken, Japan) according to the manufacturer's protocol. All cDNAs were afterward freeze at −20°C.

### Quantitative Real-Time PCR

The following *Gallus gallus* genes were examined: toll-like receptor 2 family member B (***TLR2B***), toll-like receptor 3 (***TLR3***), toll-like receptor 4 (***TLR4***), toll-like receptor adaptor molecule 1 (***TRIF***), myeloid differentiation primary response 88 (***MyD88***), interferon regulatory factor 3 (***IRF3***), nuclear factor kappa B subunit 1 (***NFkB1***), interleukin 6 (***IL6***), interleukin 8 (***IL8***), interferon-beta (***IFNW***), lipopolysaccharide-induced TNF factor (***LITAF***), transforming growth factor beta 1 (***TGFB1***), Fas cell surface death receptor (***APO-1/FAS***), *HRAS* proto-oncogene, GTPase (***HRAS***), mitogen-activated protein kinase (***MEK***), mitogen-activated protein kinase 9 (***MAPK9***), conserved helix-loop-helix ubiquitous kinase (***CHUK***), Fos proto-oncogene, *AP-1* transcription factor subunit (***FOS***), Jun proto-oncogene *AP-1* transcription factor subunit (***JUN***), phosphatidylinositol-4,5-bisphosphate 3-kinase catalytic subunit alpha (***PIK3CA***), RAC-alpha serine/threonine-protein kinase (***AKT1***), 5′-adenosine monophosphate-activated protein kinase PRKAA1 (***AMPK***), tuberous sclerosis 2 (***TSC2***), mechanistic target of rapamycin (***mTOR***), eukaryotic translation initiation factor 4E binding protein 1 (***4EBP1***) and ribosomal protein S6 kinase B1 (***S6K1***). Suitable primers were created using the GenBank sequences collected by the National Center for Biotechnology Information and U.S. National Library of Medicine (**NCBI**) except from 10 of them that they were from other mentioned references shown in Table 1. Primers were confirmed using the PRIMER BLAST algorithm for *Gallus gallus* mRNA databases to establish that there was a unique amplicon.

**Table 1:** Oligonucleotide primers used for the study of gene expression of selected targets by quantitative real-time PCR.

Target^1^	Primer sequence (5′–3′)^2^	Annealing temperature (°C)	PCR product size (bp)	GenBank (NCBI reference sequence)
*GAPDH*	F: ACTTTGGCATTGTGGAGGGT R: GGACGCTGGGATGATGTTCT	59.5	131	NM_204305.1
*ACTB*	F: CACAGATCATGTTTGAGACCTT R: CATCACAATACCAGTGGTACG	60	101	NM_205518.1
TLR signaling pathway				
*TLR2B*	F: CTTGGAGATCAGAGTTTGGA R: ATTTGGGAATTTGAGTGCTG	62	238	NM_001161650.1
*TLR3*	F: GCTTGGTTTGCTAGTTGGCT R: ACCGTGATATTTAGGCGGGG	59.5	93	NM_001011691.3
*TLR4*	F: GTCTCTCCTTCCTTACCTGCTGTTC R: AGGAGGAGAAAGACAGGGTAGGTG	64.5	187	NM_001030693.1
*TRIF*	F: TCAGCCATTCTCCGTCCTCTTC R: GGTCAGCAGAAGGATAAGGAAAGC	62	339	NM_001081506.1
*MyD88*	F: AATGGACACTGAGCTCTGCC R: CAAACCCGATCTGTGGGACA	60	126	NM_001030962.3
*IRF3*	F: GAGGATCCGGCCAAATGGAA R: GCCAAATCGTGGTGGTTGAG	60	212	NM_205372.1
*NFkB1*	F: GAAGGAATCGTACCGGGAACA R: CTCAGAGGGCCTTGTGACAGTAA	59	131	NM_205134.1
*IL6*	F: AAATCCCTCCTCGCCAATCT R: CCCTCACGGTCTTCTCCATAAA	59	106	NM_204628.1
*IL8*	F: CAGGTGACACCCGGAAGAAA R: CTGAACGTGCCTGAGCCATA	61	117	NM_205018.1
*IFNW*	F: CCTCAACCAGATCCAGCATTAC R: CCCAGGTACAAGCACTGTAGTT	60.5	167	NM_001024836.1
*LITAF*	F: GAGCAGGGCTGACACGGAT R: GCACAAAAGAGCTGATGGCAG	60	149	NM_204267.1
*TGFB1*	F: GGTTATATGGCCAACTTCTGCAT R: CCCCGGGTTGTGTTGGT	60	102	JQ423909.1
MAPK-apoptosis pathway				
*APO-1 (FAS)*	F: ACACCCGCTATTTGGGAGAC R: GAACTTGGTGGCGTTGGAGA	62	134	NM_205344.1
*HRAS*	F: GCAAGCTGAACCCACCAGAT R: TCTCCAAGTCAGGGTCCAGT	60	93	NM_205292.1
*MEK*	F: GAAGTTTGCTTCGCGTCGGG R: GGCTTCTTCTTGGGCATGTTG	62	111	NM_001005830.1
*MAPK9*	F: TTGGACTGGCAAGAACAGCG R: AAGGATAACCTCTGGCGCTC	62	86	NM_205095.1
*CHUK (IKK)*	F: TTCACTGGTAAGCTCCAGCC R: TTCTCTTGCCTCCTGCAACA	60	199	NM_001012904.1
*FOS*	F: GCCGACATGATGTACCAGGG R: GACGGGTAGTAGGTGAGGCT	62	101	NM_205508.1
*JUN*	F: CCTCCCCTGTCCCCTATTGA R: CCTTTTCCGGCATTTGGACG	61.5	99	NM_001031289.1
PI3K-Akt-mTOR pathway				
*PIK3CA*	F: ATGCCATCTTACTCCAGGCG R: TAGTCCAGAGGGACTTGGCT	61	79	NM_001004410.1
*AKT1*	F: CATCCCTTTTGTGGACCCTTCT R: CGGGAATGTCTCTTGGTGGAT	61	102	NM_205055.1
*AMPK*	F: CGGCAGATAAACAGAAGCACGAG R: CGATTCAGGATCTTCACTGCAAC	62.5	148	NM_001039603.1
*TSC2*	F: TGTGGTTCATTCGGTGTCGT R: TGGCCTCTCATTGAGGCTTG	61	149	XM_015294446.2
*mTOR*	F: GGTGATGACCTTGCCAAACT R: CTCTTGTCATCGCAACCTCA	60	220	XM_417614
*4EBP1*	F: GCGAATGTAGGTGAAGAAGAG R: AACAGGAAGGCACTCAAGG	60	146	XM_424384.5
*S6K1*	F: CAATTTGCCTCCCTACCTCA R: AAGGAGGTTCCACCTTTCGT	59.5	176	NM_001030721.1

1 *GAPDH* = glyceraldehyde 3-phosphate dehydrogenase; *ACTB* = actin, beta; *TLR2B* = toll-like receptor 2 family member B; *TLR3* = toll-like receptor 3; *TLR4* = toll-like receptor 4 (Lu et al., 2009); *TRIF* = toll-like receptor adaptor molecule 1 (Kogut et al., 2012); *MyD88* = myeloid differentiation primary response 88; *IRF3* = interferon regulatory factor 3; *NFkB1* = nuclear factor kappa B subunit 1 (Chiang et al., 2009); *IL6* = interleukin 6 (Wang et al., 2012); *IL8* = interleukin 8; *IFNW* = interferon beta (Jie et al., 2013); *LITAF* = lipopolysaccharide-induced TNF Factor; *TGFB1* = transforming growth factor beta 1 (Sweeney et al., 2016); *APO-1/FAS* = Fas cell surface death receptor; *HRAS* = HRas proto-oncogene, GTPase; *MEK* = mitogen-activated protein kinase; *MAPK9* = mitogen-activated protein kinase 9; *CHUK* = conserved helix-loop-helix ubiquitous kinase; *FOS* = Fos proto-oncogene, AP-1 transcription factor subunit; *JUN* = Jun proto-oncogene AP-1 transcription factor subunit; *PIK3CA* = phosphatidylinositol-4,5-bisphosphate 3-kinase catalytic subunit alpha; *AKT1* = RAC-alpha serine/threonine-protein kinase; *AMPK* = 5′-adenosine monophosphate-activated protein kinase PRKAA1; *TSC2* = tuberous sclerosis 2; *mTOR* = mechanistic target of rapamycin (Chen et al., 2016); *4EBP1* = eukaryotic translation initiation factor 4E binding protein 1 (Chang et al., 2015); *S6K1* = ribosomal protein S6 kinase B1 (Chang et al., 2015)

2 F: forward, R: reverse.

Real-time PCR was accomplished in 96 well microplates with a SaCycler-96 Real-Time PCR System (Sacace Biotechnologies s.r.l., Como, Italy) and FastGene IC Green 2× qPCR universal mix (Nippon Genetics, Tokyo, Japan). Each reaction included 12.5 ng RNA equivalents as well as 200 nmol/L of forward and reverse primers for each gene. The reactions were hatched at 95°C for 3 min, followed by 40 cycles of 95°C for 5 s 59.5 to 62°C (depends on the target gene) for 20 s, 72°C for 33 s. This was pursued by a melt curve analysis to regulate the reaction specificity. Each sample was determined in duplicates. Relative expression ratios of target genes were calculated according to Pfaffl et al. (2001) and Hellemans et al. (2007) using GAPDH and ACTB as reference genes (Pfaffl, 2001; Hellemans et al., 2007).

### Statistical Analysis

Experimental data were tested for normality using the Kolmogorov-Smirnov test and found to be normally distributed. Data were analyzed with the general linear model (**GLM**)—general factorial ANOVA procedure using diet type (L, H) and PFA addition (− and +) as fixed factors. Statistically significant effects were further analyzed and means were compared using Tukey's honestly significant difference multiple comparison procedure. Statistical significance was determined at *P* ≤ 0.05. All statistical analyses were done using the SPSS for Windows Statistical Package Program (SPSS 17.0, Inc., Chicago, IL).

## RESULTS

### Growth Performance Responses

Diet type had a significant effect on the overall broiler growth performance responses. In particular, overall body weight gain and overall FCR were better in chickens fed diet type H with 100% ME and CP compared to the 95% ME and CP type diet (L). The addition of PFA significantly improved overall body weight gain and overall FCR in the reduced ME and CP specification diet type (L), as stated in detail by Griela et al., 2021

### Relative Expression of the Critical Genes Studied

The gene expression results have been presented starting from the significant factor interactions (diet type × PFA supplementation) followed by significant diet type and PFA effects per intestinal segment and have been grouped in 2 tables per segment based on the pathway involved.

### Duodenum

In the duodenum the relative expressions of the genes studied are listed in Tables 2 and 3. A significant interaction of diet type and PFA supplementation was shown only for the *mTOR* gene (*P*_D×P_ = 0.040) with the broilers in treatment H− displaying the highest expression levels compared to the other treatments (data not shown). Compared to the recommended 100% ME and CP specs, the reduced 95% specs diet resulted in upregulation of *myD88* (*P*_D_ = 0.035) and *PIK3CA* (*P*_D_ < 0.001) and downregulation of *APO1* (*P*_D_ = 0.015) and *mTOR* (*P*_D_ = 0.021) in the duodenum (Tables 2 and 3). With respect to PFA effects, PFA supplementation reduced the expression of *TLR2B* (*P*_P_ = 0.012), *MyD88* (*P*_P_ = 0.009), *IL8* (*P*_P_ = 0.017), while it increased the expression of *HRAS* (*P*_P_ = 0.031).

**Table 2:** Relative gene expression of toll-like receptors (TLR2B, TLR3, TLR4), adaptor molecules (TRIF, MyD88), transcription factors (IRF3, NFkB1), interleukin 6 (IL6), interleukin 8 (IL8), interferon-beta (IFNW), lipopolysaccharide-induced TNF factor (LITAF), and transforming growth factor beta 1 (TGF) in duodenal mucosa of 42-day-old broilers.

		Type of diet^1^		PFA supplementation^2^			*P* values^3^		
Duodenum	Gene	L	H	No	Yes	SEM^4^	Diet (D)	PFA (P)	D × P
TLR signaling pathway	*TLR2B*	1.64	1.01	1.88^x^	0.77^y^	0.343	0.092	0.012	0.060
	*TLR3*	1.28	1.00	1.19	1.09	0.170	0.112	0.562	0.781
	*TLR4*	1.33	1.17	1.33	1.18	0.274	0.562	0.589	0.719
	*TRIF*	1.13	1.03	1.20	0.97	0.132	0.466	0.093	0.481
	*MyD88*	1.30^a^	0.97^b^	1.34^X^	0.93^Y^	0.150	0.035	0.009	0.263
	*IRF3*	1.08	1.43	1.24	1.27	0.327	0.288	0.925	0.965
	*NFkB1*	1.15	0.98	1.12	1.01	0.129	0.185	0.395	0.585
	*IL6*	1.29	0.93	0.98	1.24	0.207	0.084	0.227	0.573
	*IL8*	1.19	0.96	1.41^x^	0.74^y^	0.265	0.406	0.017	0.161
	*IFNW*	1.12	1.08	1.19	1.02	0.149	0.573	0.294	0.450
	*LITAF*	1.46	1.26	1.40	1.32	0.257	0.437	0.761	0.110
	*TGF*	1.28	0.99	1.20	1.07	0.182	0.125	0.491	0.633

1 Two diet types: L (formulated at 95% of optimum broiler metabolizable energy and crude protein requirements) and H (formulated at 100% of optimum broiler metabolizable energy and crude protein).

2 Phytogenic supplementation (No = 0 mg/kg diet and Yes = 150 mg/kg diet). Data shown for PFA represent means from 18 replicate pens (e.g., 9 + 9 for no PFA supplementation treatments L− and H− and 9 + 9 for PFA supplementation treatments L+ and H+).

3 Within the same row means with different superscript per diet type (a, b or A, B), and phytogenic (x, y or X, Y) differ significantly (*P* < 0.05 or 0.01).

4 Pooled standard error of means.

**Table 3:** Relative gene expression of apoptosis-related genes (APO1, HRAS, MEK, MAPK9, CHUK, FosB, and JunD) and metabolism-related genes (PIK3CA, AKT1, AMPK, TSC2, mTOR, 4EBP1, and S6K1) in duodenal mucosa of 42-day-old broilers.

		Type of diet^1^		PFA supplementation^2^			*P* values^3^		
Duodenum	Gene	L	H	No	Yes	SEM^4^	Diet (D)	PFA (P)	D × P
MAPK pathway	*APO1*	0.89^b^	1.25^a^	1.05	1.10	0.138	0.015	0.723	0.952
	*HRAS*	1.11	1.15	0.91^y^	1.35^x^	0.194	0.833	0.031	0.720
	*MEK*	0.98	1.11	*0.95*	*1.14*	0.098	0.188	0.065	0.858
	*MAPK9*	1.22	1.01	1.26	0.97	0.181	0.252	0.123	0.858
	*CHUK*	1.18	1.00	1.11	1.06	0.150	0.228	0.738	0.281
	*FOS*	1.16	1.36	1.46	1.06	0.325	0.551	0.221	0.384
	*JUN*	0.97	1.21	1.04	1.14	0.153	0.132	0.538	0.360
PI3K-Akt-mTOR signaling pathway	*PIK3CA*	1.99^A^	0.92^B^	1.67	1.24	0.277	<0.001	0.131	0.095
	*AKT1*	1.09	1.04	1.04	1.09	0.141	0.758	0.728	0.966
	*AMPK*	1.12	1.06	1.06	1.13	0.177	0.729	0.706	0.496
	*TSC2*	2.02	1.31	1.91	1.42	0.444	0.118	0.280	0.841
	*mTOR*	0.98^b^	1.23^a^	1.08	1.13	0.105	0.021	0.592	0.040
	*4EBP1*	1.14	1.00	0.98	1.16	0.132	0.304	0.184	0.468
	*S6K1*	1.00	1.13	1.02	1.11	0.153	0.409	0.569	0.883

1 Two diet types: L (formulated at 95% of optimum broiler metabolizable energy and crude protein requirements) and H (formulated at 100% of optimum broiler metabolizable energy and crude protein).

2 Phytogenic supplementation (No = 0 mg/kg diet and Yes = 150 mg/kg diet). Data shown for PFA represent means from 18 replicate pens (e.g., 9 + 9 for no PFA supplementation treatments L− and H− and 9 + 9 for PFA supplementation treatments L+ and H+).

3 Within the same row means with different superscript per diet type (a, b or A, B), and phytogenic (x, y or X, Y) differ significantly (*P* < 0.05 or 0.01).

4 Pooled standard error of means.

### Jejunum

In the jejunum, the relative expressions of the genes studied are listed in Tables 4 and 5. A significant interaction of diet type and PFA supplementation was shown only for the *IL8* (*P*_D×P_ = 0.030), with treatment H- showing the highest expression levels compared to the other 3 treatments (data not shown). Compared to the recommended 100% ME and CP specs, the reduced 95% specs diet resulted in upregulation of *TLR4* (*P*_D_ = 0.004), *NFkB1* (*P*_D_ = 0.022), and *IL6* (*P*_D_ = 0.021) and downregulation of *FOS* (*P*_D_ = 0.022) in the jejunum (Tables 4 and 5). The PFA supplementation reduced the jejunal expression of *IL8* (*P*_P_ = 0.001), *APO1* (*P*_P_ = 0.015), and *FOS* (*P*_P_ = 0.029).

**Table 4:** Relative gene expression of toll-like receptors (TLR2B, TLR3, TLR4), adaptor molecules (TRIF, MyD88), transcription factors (IRF3, NFkB1), interleukin 6 (IL6), interleukin 8 (IL8), interferon-beta (IFNW), lipopolysaccharide-induced TNF factor (LITAF), and transforming growth factor beta 1 (TGF) in jejunal mucosa of 42-day-old broilers.

		Type of diet^1^		PFA supplementation^2^			*P* values^3^		
Jejunum	Gene	L	H	No	Yes	SEM^4^	Diet (D)	PFA (P)	D × P
TLR signaling pathway	*TLR2B*	1.26	1.30	1.55	1.02	0.355	0.916	0.143	0.712
	*TLR3*	1.38	1.16	1.46	1.08	0.163	0.355	0.111	0.293
	*TLR4*	1.53^A^	0.85^B^	1.27	1.11	0.219	0.004	0.489	0.483
	*TRIF*	1.09	0.98	1.02	1.06	0.094	0.427	0.750	0.918
	*MyD88*	1.33	0.94	1.18	1.09	0.136	0.054	0.669	0.127
	*IRF3*	1.18	1.14	1.27	1.05	0.220	0.885	0.343	0.865
	*NFkB1*	1.18^a^	0.94^b^	1.16	0.96	0.100	0.022	0.060	0.643
	*IL6*	1.54^a^	0.95^b^	1.30	1.19	0.246	0.021	0.670	0.414
	*IL8*	1.33	1.39	1.85^X^	0.87^Y^	0.195	0.808	0.001	0.030
	*IFNW*	1.01	1.28	1.09	1.20	0.099	0.058	0.415	0.239
	*LITAF*	1.46	1.15	1.52	1.10	0.287	0.286	0.156	0.601
	*TGF*	1.17	1.12	1.21	1.08	0.211	0.831	0.539	0.614

1 Two diet types: L (formulated at 95% of optimum broiler metabolizable energy and crude protein requirements) and H (formulated at 100% of optimum broiler metabolizable energy and crude protein).

2 Phytogenic supplementation (No = 0 mg/kg diet and Yes = 150 mg/kg diet). Data shown for PFA represent means from 18 replicate pens (e.g., 9 + 9 for no PFA supplementation treatments L− and H− and 9 + 9 for PFA supplementation treatments L+ and H+).

3 Within the same row means with different superscript per diet type (a, b or A, B), and phytogenic (x, y or X, Y) differ significantly (*P* < 0.05 or 0.01).

4 Pooled standard error of means.

**Table 5:** Relative gene expression of apoptosis-related genes (APO1, HRAS, MEK, MAPK9, CHUK, FosB, and JunD) and metabolism-related genes (PIK3CA, AKT1, AMPK, TSC2, mTOR, 4EBP1, and S6K1) in jejunal mucosa of 42-day-old broilers.

		Type of diet^1^		PFA supplementation^2^			*P* values^3^		
Jejunum	Gene	L	H	No	Yes	SEM^4^	Diet (D)	PFA (P)	D × P
MAPK pathway	*APO1*	1.01	1.16	1.29^x^	0.89^y^	0.156	0.326	0.015	0.399
	*HRAS*	1.07	1.08	1.15	0.99	0.132	0.953	0.233	0.920
	*MEK*	1.14	1.13	1.24	1.03	0.183	0.974	0.253	0.789
	*MAPK9*	1.12	1.07	1.23	0.96	0.157	0.793	0.089	0.496
	*CHUK*	1.10	1.03	1.08	1.05	0.124	0.593	0.796	0.587
	*FOS*	0.94^b^	1.52^a^	1.50^x^	0.95^y^	0.240	0.022	0.029	0.095
	*JUN*	1.03	1.21	1.25	0.99	0.179	0.343	0.157	0.241
PI3K-Akt-mTOR signaling pathway	*PIK3CA*	1.25	1.05	1.29	1.01	0.211	0.345	0.199	0.855
	*AKT1*	1.13	1.04	1.12	1.04	0.144	0.559	0.574	0.514
	*AMPK*	1.23	1.02	1.26	0.99	0.163	0.218	0.113	0.200
	*TSC2*	1.31	0.98	1.21	1.08	0.224	0.149	0.583	0.499
	*mTOR*	1.10	1.02	1.12	1.00	0.091	0.526	0.389	0.494
	*4EBP1*	0.99	1.17	1.18	0.99	0.143	0.224	0.194	0.812
	*S6K1*	1.06	1.09	1.13	1.02	0.140	0.853	0.453	0.890

1 Two diet types: L (formulated at 95% of optimum broiler metabolizable energy and crude protein requirements) and H (formulated at 100% of optimum broiler metabolizable energy and crude protein).

2 Phytogenic supplementation (No = 0 mg/kg diet and Yes = 150 mg/kg diet). Data shown for PFA represent means from 18 replicate pens (e.g., 9 + 9 for no PFA supplementation treatments L− and H− and 9+9 for PFA supplementation treatments L+ and H+).

3 Within the same row means with different superscript per diet type (a, b or A, B), and phytogenic (x, y or X, Y) differ significantly (*P* < 0.05 or 0.01).

4 Pooled standard error of mean.

### Ileum

In the ileum, the relative expressions of the genes studied are listed in Tables 6 and 7. A significant interaction of diet type and PFA supplementation was shown only for the *HRAS* gene (*P*_D×P_ = 0.005). In particular, higher expression levels of *HRAS* were shown in the broilers of treatment L- compared to the other 3 treatments (data not shown). Compared to the recommended 100% ME and CP specs, the reduced 95% specs diet resulted in upregulation of *TLR3* (*P*_D_ = 0.048), *TRIF* (*P*_D_ = 0.046), *MyD88* (*P*_D_ = 0.001), *APO1* (*P*_D_ = 0.023), *CHUK* (*P*_D_ = 0.001), *AMPK* (*P*_D_ = 0.026), *TSC2* (*P*_D_ < 0.001), and *mTOR* (*P*_D_ < 0.001) in the ileum (Tables 6 and 7). The PFA supplementation reduced the ileal expression of *LITAF* (*P*_P_ = 0.047).

**Table 6:** Relative gene expression of toll-like receptors (TLR2B, TLR3, TLR4), adaptor molecules (MyD88, TRIF), transcription factors (IRF3, NFkB1), interleukin 6 (IL6), interleukin 8 (IL8), interferon-beta (IFNW), lipopolysaccharide-induced TNF factor (LITAF), and transforming growth factor beta 1 (TGF) in ileal mucosa of 42-day-old broilers.

		Type of diet^1^		PFA supplementation^2^			*P* values^3^		
Ileum	Gene	L	H	No	Yes	SEM^4^	Diet (D)	PFA (P)	D × P
TLR signaling pathway	*TLR2B*	1.64	0.91	1.53	1.02	0.391	0.068	0.850	0.252
	*TLR3*	1.35^a^	0.96^b^	1.28	1.02	0.190	0.048	0.195	0.241
	*TLR4*	1.07	1.20	1.25	1.03	0.189	0.481	0.252	0.733
	*TRIF*	1.19^a^	0.93^b^	1.08	1.05	0.124	0.046	0.820	0.364
	*MyD88*	1.39^A^	0.85^B^	1.20	1.03	0.143	0.001	0.252	0.797
	*IRF3*	1.18	1.46	1.36	1.29	0.343	0.414	0.845	0.355
	*NFkB1*	1.21	0.98	1.12	1.06	0.149	0.135	0.682	0.210
	*IL6*	1.27	1.08	1.35	1.00	0.233	0.408	0.147	0.111
	*IL8*	1.02	1.09	1.04	1.08	0.158	0.750	0.845	0.673
	*IFNW*	1.05	1.03	1.03	1.05	0.083	0.836	0.880	0.085
	*LITAF*	1.59	1.43	1.91^x^	1.12^y^	0.381	0.668	0.047	0.798
	*TGF*	1.41	1.01	1.29	1.13	0.309	0.202	0.602	0.477

1 Two diet types: L (formulated at 95% of optimum broiler metabolizable energy and crude protein requirements) and H (formulated at 100% of optimum broiler metabolizable energy and crude protein).

2 Phytogenic supplementation (No = 0 mg/kg diet and Yes = 150 mg/kg diet). Data shown for PFA represent means from 18 replicate pens (e.g., 9 + 9 for no PFA supplementation treatments L− and H− and 9 + 9 for PFA supplementation treatments L+ and H+).

3 Within the same row means with different superscript per diet type (a, b or A, B), and phytogenic (x, y or X, Y) differ significantly (*P* < 0.05 or 0.01).

4 Pooled standard error of mean.

**Table 7:** Relative gene expression of apoptosis-related genes (APO1, HRAS, MEK, MAPK9, CHUK, FosB, and JunD) and metabolism-related genes (PIK3CA, AKT1, AMPK, TSC2, mTOR, 4EBP1, and S6K1) in ileal mucosa of 42-day-old broilers.

		Type of diet^1^		PFA supplementation^2^			*P* values^3^		
Ileum	Gene	L	H	No	Yes	SEM^4^	Diet (D)	PFA (P)	D × P
MAPK pathway	*APO1*	1.23^a^	0.92^b^	1.09	1.06	0.129	0.023	0.814	0.299
	*HRAS*	1.11	1.03	1.08	1.05	0.120	0.514	0.815	0.005
	*MEK*	0.97	1.34	1.09	1.22	0.241	0.134	0.595	0.089
	*MAPK9*	1.18	0.99	1.08	1.10	0.143	0.189	0.902	0.586
	*CHUK*	1.40^A^	0.89^B^	1.22	1.07	0.140	0.001	0.275	0.403
	*FOS*	1.56	1.14	1.35	1.35	0.363	0.254	0.984	0.426
	*JUN*	1.36	0.99	1.20	1.16	0.233	0.125	0.859	0.635
PI3K-Akt-mTOR signaling pathway	*PIK3CA*	1.14	1.06	1.20	1.01	0.166	0.640	0.254	0.206
	*AKT1*	1.02	1.15	0.99	1.17	0.142	0.375	0.214	0.620
	*AMPK*	1.40^a^	0.92^b^	1.23	1.09	0.201	0.026	0.509	0.523
	*TSC2*	2.49^A^	1.02^B^	1.74	1.77	0.307	<0.001	0.941	0.902
	*mTOR*	1.41^A^	0.84^B^	1.09	1.17	0.123	<0.001	0.482	0.113
	*4EBP1*	1.10	1.08	1.06	1.13	0.157	0.911	0.659	0.736
	*S6K1*	1.12	0.98	1.09	1.01	0.106	0.191	0.421	0.162

1 Two diet types: L (formulated at 95% of optimum broiler metabolizable energy and crude protein requirements) and H (formulated at 100% of optimum broiler metabolizable energy and crude protein).

2 Phytogenic supplementation (No = 0 mg/kg diet and Yes = 150 mg/kg diet). Data shown for PFA represent means from 18 replicate pens (e.g., 9 + 9 for no PFA supplementation treatments L− and H− and 9 + 9 for PFA supplementation treatments L+ and H+).

3 Within the same row means with different superscript per diet type (a, b or A, B), and phytogenic (x, y or X, Y) differ significantly (*P* < 0.05 or 0.01).

4 Pooled standard error of mean.

### Ceca

In the ceca, the relative expressions of the genes studied are listed in Tables 8 and 9. A significant diet type and PFA supplementation was shown only for the *IL8* gene (*P*_D×P_ = 0.022), with broilers in treatment L− displaying the highest expression levels compared to the other 3 treatments (data not shown). Compared to the recommended 100% ME and CP specs, the reduced 95% specs diet resulted in upregulation of *TRIF* (*P*_D_ = 0.031), *IL6* (*P*_D_ = 0.022), *IL8* (*P*_D_ = 0.003), *IFNW* (*P*_D_ = 0.016), *TGF* (*P*_D_ = 0.003), *MAPK9* (*P*_D_ = 0.021), *CHUK* (*P*_D_ = 0.012), *TSC2* (*P*_D_ = 0.009) and downregulation of *4EBP1* (*P*_D_ = 0.011) in the ceca (Tables 8 and 9). PFA supplementation reduced the cecal expression of *TLR3* (*P*_P_ = 0.012), *MyD88* (*P*_P_ = 0.040), *TSC2* (*P*_P_ = 0.042), while it increased the expression of *4EBP1* (*P*_P_ < 0.001).

**Table 8:** Relative gene expression of toll-like receptors (TLR2B, TLR3, TLR4), adaptor molecules (MyD88, TRIF), transcription factors (IRF3, NFkB1), interleukin 6 (IL6), interleukin 8 (IL8), interferon-beta (IFNW), lipopolysaccharide-induced TNF factor (LITAF), and transforming growth factor beta 1 (TGF) in cecal mucosa of 42-day-old broilers.

		Type of diet^1^		PFA supplementation^2^			*P* values^3^		
Cecum	Gene	L	H	No	Yes	SEM^4^	Diet (D)	PFA (P)	D × P
TLR signaling pathway	*TLR2B*	1.53	0.98	1.43	1.09	0.311	0.086	0.281	0.658
	*TLR3*	1.25	1.11	1.43^x^	0.93^y^	0.186	0.461	0.012	0.124
	*TLR4*	1.35	0.97	1.20	1.13	0.217	0.085	0.749	0.573
	*TRIF*	1.24^a^	0.93^b^	1.10	1.08	0.137	0.031	0.917	0.138
	*MyD88*	1.49	0.81	1.26^x^	1.04^y^	0.181	0.300	0.040	0.126
	*IRF3*	1.43	1.22	1.40	1.25	0.347	0.545	0.663	0.136
	*NFkB1*	1.22	0.98	1.16	1.04	0.186	0.208	0.528	0.127
	*IL6*	1.54^a^	0.90^b^	1.21	1.24	0.266	0.022	0.911	0.504
	*IL8*	1.54^A^	0.73^B^	1.26	1.01	0.254	0.003	0.330	0.022
	*IFNW*	1.35^a^	0.90^b^	1.20	1.05	0.175	0.016	0.407	0.557
	*LITAF*	1.61	1.36	1.58	1.39	0.357	0.483	0.607	0.292
	*TGF*	1.81^A^	0.84^B^	1.30	1.34	0.299	0.003	0.889	0.880

1 Two diet types: L (formulated at 95% of optimum broiler metabolizable energy and crude protein requirements) and H (formulated at 100% of optimum broiler metabolizable energy and crude protein).

2 Phytogenic supplementation (No = 0 mg/kg diet and Yes = 150 mg/kg diet). Data shown for PFA represent means from 18 replicate pens (e.g., 9 + 9 for no PFA supplementation treatments L− and H− and 9 + 9 for PFA supplementation treatments L+ and H+).

3 Within the same row means with different superscript per diet type (a, b or A, B), and phytogenic (x, y or X, Y) differ significantly (*P* < 0.05 or 0.01).

4 Pooled standard error of mean.

**Table 9:** Relative gene expression of apoptosis-related genes (APO1, HRAS, MEK, MAPK9, CHUK, FosB, and JunD) and metabolism-related genes (PIK3CA, AKT1, AMPK, TSC2, mTOR, 4EBP1, and S6K1) in cecal mucosa of 42-day-old broilers.

		Type of diet^1^		PFA supplementation^2^			*P* values^3^		
Cecum	Gene	L	H	No	Yes	SEM^4^	Diet (D)	PFA (P)	D × P
MAPK pathway	*APO1*	1.12	1.10	1.20	1.02	0.187	0.892	0.346	0.533
	*HRAS*	1.23	1.04	1.02	1.25	0.207	0.362	0.277	0.682
	*MEK*	1.09	1.26	1.22	1.13	0.228	0.448	0.668	0.066
	*MAPK9*	1.52^a^	1.00^b^	1.27	1.26	0.213	0.021	0.975	0.147
	*CHUK*	1.49^a^	0.91^b^	1.27	1.12	0.216	0.012	0.477	0.912
	*FOS*	0.98	0.84	1.01	0.81	0.209	0.511	0.333	0.291
	*JUN*	1.14	1.14	1.09	1.20	0.212	0.992	0.608	0.872
PI3K-Akt-mTOR signaling pathway	*PIK3CA*	1.33	1.02	1.21	1.15	0.228	0.180	0.789	0.733
	*AKT1*	1.15	1.07	1.07	1.14	0.176	0.637	0.691	0.776
	*AMPK*	1.14	1.12	1.12	1.14	0.208	0.920	0.899	0.509
	*TSC2*	1.69^A^	0.91^B^	1.60^x^	1.00^y^	0.279	0.009	0.042	0.868
	*mTOR*	1.12	1.05	1.06	1.11	0.117	0.568	0.627	0.374
	*4EBP1*	1.64^b^	2.62^a^	1.43^Y^	2.83^X^	0.360	0.011	<0.001	0.054
	*S6K1*	1.66	1.46	1.67	1.45	0.200	0.319	0.281	0.208

1 Two diet types: L (formulated at 95% of optimum broiler metabolizable energy and crude protein requirements) and H (formulated at 100% of optimum broiler metabolizable energy and crude protein).

2 Phytogenic supplementation (No = 0 mg/kg diet and Yes = 150 mg/kg diet). Data shown for PFA represent means from 18 replicate pens (e.g., 9 + 9 for no PFA supplementation treatments L− and H− and 9 + 9 for PFA supplementation treatments L+ and H+).

3 Within the same row means with different superscript per diet type (a, b or A, B), and phytogenic (x, y or X, Y) differ significantly (*P* < 0.05 or 0.01).

4 Pooled standard error of mean.

## DISCUSSION

Nutritional solutions that can support zootechnical performance at reduced dietary specifications could effectively support the broiler industry, especially nowadays that feed costs continuously increase. In this respect various feed additives are actively researched for their direct and indirect effects on broiler performance and gut function and health such as enzymes, acidifiers, probiotics, prebiotics, and phytogenics (Yang et al., 2009; Hussein et al., 2019; Mountzouris et al., 2019; Pearlin et al., 2020; Rehman et al., 2020; Swaggerty et al., 2020; Rafeeq et al., 2022). However, it is evident that a deeper understanding of the effects of dietary energy and protein levels on critical broiler homeostatic responses such as those related with inflammation control, cellular apoptosis, nutrient sensing and protein synthesis is still warranted.

It was previously shown that reduction of the dietary broiler hybrid recommendations for ME and CP by 5%, reduced broiler performance but also induced an antioxidant response via the *Nrf2* pathway (Griela et al., 2021). Dietary phytogenic applications may ameliorate the negative effects of reduced dietary specifications on broiler performance and likewise increase the intestinal total antioxidant capacity (Bravo et al., 2014; Paraskeuas et al., 2016; Mountzouris et al., 2020; Griela et al., 2021).

**Figure 1:**
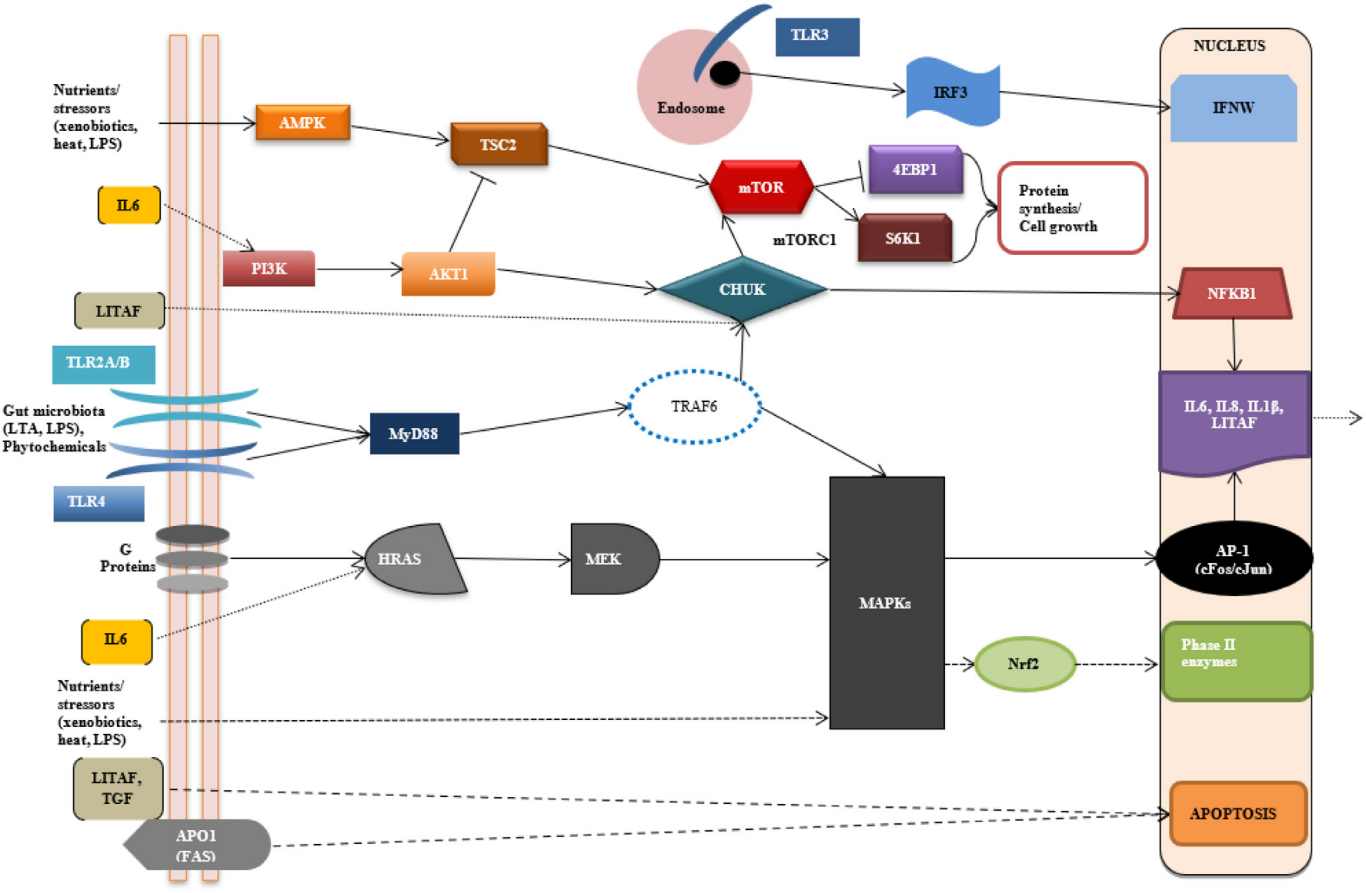
Different signaling pathways link TLRs to MAPK and mTOR in broilers. *TLR3* through *TRIF* (MyD88-independent pathway) activates the IRF3-dependent secretion of *IFNW. TLR2* and *TLR4* stimulate, through MyD88-dependent pathway, the *CHUK* and *MAPK9* which trigger *NFkB1* and AP-1(*Fos*/*Jun*) respectively that contribute to subsequent production of proinflammatory cytokines. In this pathway are also involved *PI3K* and *AKT1* which trigger *CHUK* to stimulate *mTOR* pathway and inhibit *TSC2* which induced by *AMPK*. Also, G-proteins activate *HRAS* and through MAPK pathway they induce Fos/Jun dimer to express proinflammatory cytokines. MAPK pathways induce ARE-mediated gene expression with the stimulation of *Nrf2*. Initiated *IL6* signaling can activate *HRAS* and PI3K/AKT signaling pathways. *TNF-α* as well as *TGF* and *APO1* induce apoptosis, whereas *LITAF* also activates *NFkB1* and expression of prosurvival genes. LTA = lipoteichoic acid; LPS = lipopolysaccharide; *TLR2* = toll-like receptor 2; *TLR3* = toll-like receptor 3; *TLR4* = toll-like receptor 4; *TRIF* = toll-like receptor adaptor molecule 1; *MyD88* = myeloid differentiation primary response 88; *IRF3* = interferon regulatory factor 3; *NFkB1* = nuclear factor kappa B subunit 1; *IL6* = interleukin 6; *IL8* = interleukin 8; *IFNW* = interferon beta; *LITAF* = lipopolysaccharide-induced TNF Factor; *TGF* = transforming growth factor; *APO-1/FAS* = Fas cell surface death receptor; *HRAS* = HRas proto-oncogene, GTPase; *MEK* = mitogen-activated protein kinase; *MAPK* = mitogen-activated protein kinase; *CHUK* = conserved helix-loop-helix ubiquitous kinase; *FOS* = Fos proto-oncogene, AP-1 transcription factor subunit; *JUN* = Jun proto-oncogene AP-1 transcription factor subunit; *PIK3* = phosphatidylinositol-4,5-bisphosphate 3-kinase; *AKT1* = RAC-alpha serine/threonine-protein kinase; *AMPK* = 5′-adenosine monophosphate-activated protein kinase PRKAA1; *TSC2* = tuberous sclerosis 2; *mTOR* = mechanistic target of rapamycin; *4E-BP1* = eukaryotic translation initiation factor 4E binding protein 1; *S6K1* = ribosomal protein S6 kinase B1.

This study has aimed to delve further into the mechanisms underlying dietary and PFA effects on broiler performance. In particular, critical gene components of pathways regulating inflammation control (*TLR* pathway), cellular apoptosis (*MAPK* pathway) and nutrient sensing and metabolism (*PI3KCA/Akt/mTOR* pathway) were studied along the broiler intestinal mucosa. For ease, a simplified presentation of the major biomarkers that involve the key cellular pathways stated above is shown in Figure 1 and explained below.

There are several associations between genes from the *TLR* signaling pathway and the *MAPK* pathway. In particular, *TLR2* recruits and upregulates through *MyD88* the IKK complex (*IKKα*/*IKKβ*) and *MAPK9* (*JNK*). In turn, the encoded enzymes stimulate *NFkB/AP-1*-dependent expression of *Fos*/*Jun* and the production of *IL6, IL8*, and *LITAF* that all contribute to cell death (Gomard et al., 2010). Moreover, the *TLR3* activates through *TRIF* the *IRF3*-dependent secretion of *IFN*-beta (Gao et al., 2021). In addition, *TLR4* may use *Fos*/*Jun* (*AP-1*) and the autocrine secretion of *LITAF* and *IFN*-beta to enhance cell death (Salaun et al., 2007). Finally, G-proteins activate *HRAS* and through *MAPK* pathway they induce *Fos*/*Jun* dimer to express proinflammatory cytokines such as *IL6* and *IL8* (Agalakova and Gusev, 2012). Finally, the *PI3KCA*/*Akt*/*mTOR* pathway assembles 2 multiprotein complexes, *mTORC1* and *mTORC2*, which are constructed of individual protein binding partners to adjust cell growth and metabolism. The *PI3K* signaling pathway has an important role in nutrient sensing cell growth, proliferation and survival (Yang et al., 2019).

**Figure 2:**
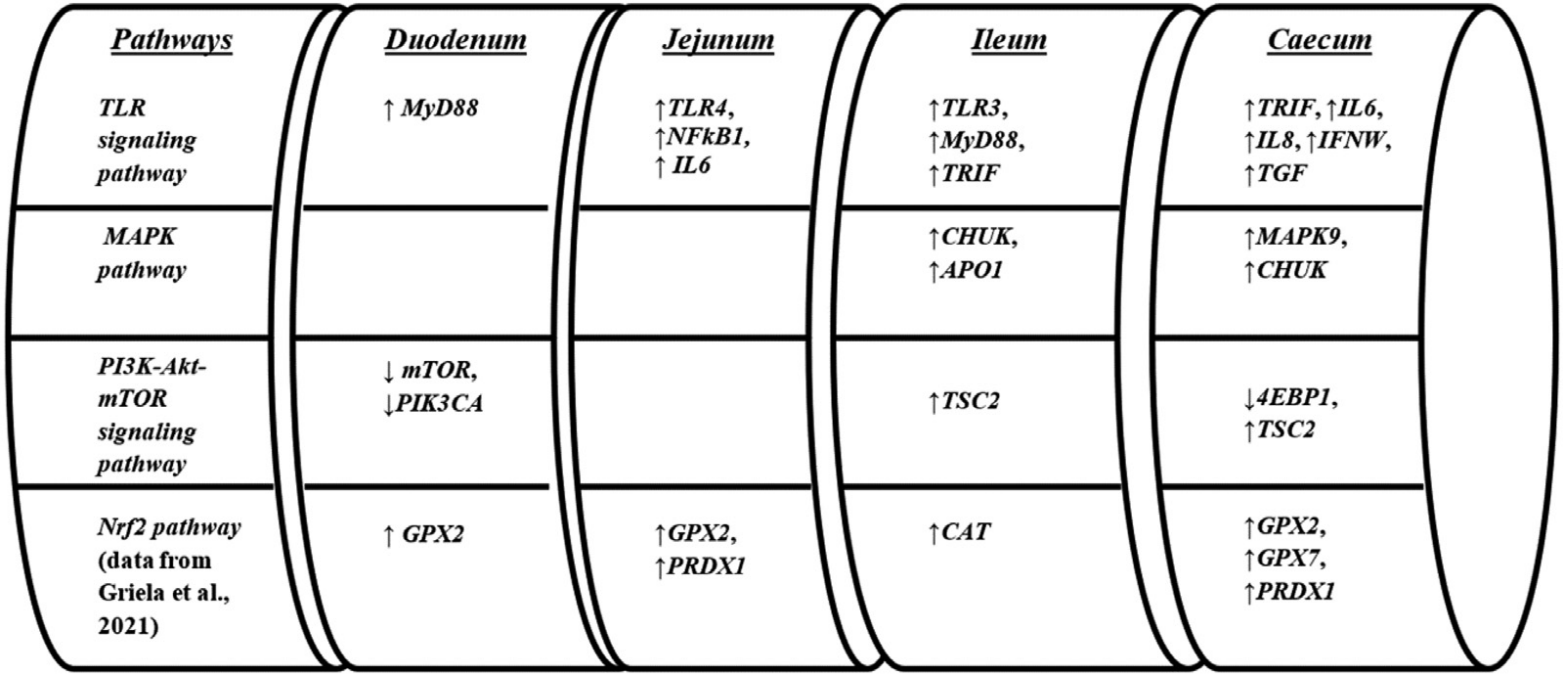
Effects of L diets compared to H diets on TLR signaling pathway, MAPK pathway PI3K-Akt-mTOR signaling pathway and Nrf2 pathway (data from Griela et al., 2021) related genes throughout the intestine.

The limited number of interactions found for only 3 (i.e., *mTOR, IL8*, and *HRAS*) of the total genes studied, attenuated the importance of concurrent effects initially expected between diet type and PFA inclusion. On the other hand, diet type modulated the *TLR* signaling to inflammation genes at an extent ranging from 8 to 42% of the genes studied, depending on the intestinal segment. Among the components that were modulated by diet type were *TLR3* and *TLR4*, adaptor molecules *MyD88* and *TRIF*, nuclear factor *NFkB1*, the proinflammatory cytokines *IL6* and *IL8*, the interferon *IFNW* and the *TGF* factor. In particular, compared to the 100% ME and CP diet type (H), it was shown that the 95% spec diet resulted in upregulating all of the above stated gene transcripts (Figure 2). The latter findings suggested that the reduced spec diet is concomitant with regulating the homeostatic inflammatory control baseline at a higher physiological-functional level compared with the 100% spec diet. Although not directly relevant, lower protein intake has also resulted in upregulated inflammatory response and oxidative stress in humans (Hruby and Jacques, 2019). In addition, compared to the 100% ME and CP diet type, the 95% spec diet upregulated significant biomarkers (i.e., *CHUK, MAPK9, APO1*) of the *MAPK* pathway that are linked with cellular apoptosis. The latter finding may suggest that a reduced intake of ME and CP may regulate the homeostatic control of cellular apoptosis at a “faster” rate. Despite the fact that there were not directly comparable studies, in a mice study it was shown that energy restriction increased lipid peroxidation, inflammation and apoptosis in G93A mice (Patel et al., 2010). Moreover, the 95% spec diet resulted in modulation of critical genes related to mTOR, noted by reduced expression of the *mTOR, PI3K*, and *4EBP1* and increased expression of *TSC2*, compared to the 100% type diet. The *mTOR* is known to sense nutrients and energy and instruct the cells to increase their work capacity and ATP production (Laplante and Sabatini, 2012). Therefore, the finding above indicates that the reduced energy and protein intake in the case of 95% ME and CP diet specs got sensed by *mTOR* that in turn may have signaled cells to retard rapid growth, which in this study became evident as reduced broiler growth performance.

**Figure 3:**
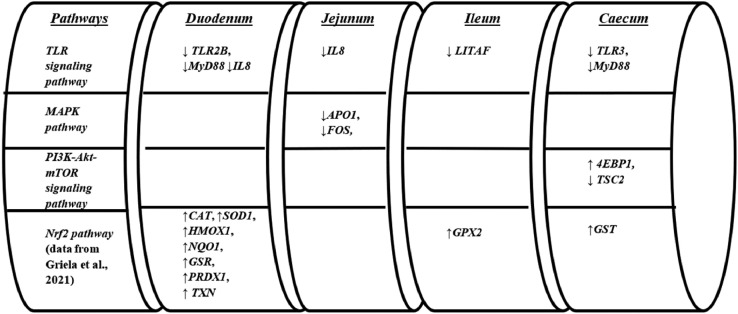
Beneficial effects of PFA supplementation on TLR signaling pathway, *MAPK* pathway *PI3K*-*Akt*-*mTOR* signaling pathway and Nrf2 pathway (data from Griela et al., 2021) related genes throughout the intestine.

On the other hand, phytogenic inclusion modulated the *TLR* signaling to inflammation genes to an extent ranging from 8 to 25% of the genes studied, depending on the intestinal segment. Among the components modulated were *TLR2B* and *TLR3*, adaptor molecule *MyD88* and the proinflammatory cytokines *IL8* and *TNFa*. The results above were in line with several other studies reporting relevant effects for PFA supplementation (Lu et al., 2014; Paraskeuas and Mountzouris, 2019; Wang et al., 2021). In particular, PFA inclusion, compared to the non-inclusion, downregulated all the above gene transcripts (Figure 3). This finding may suggest that PFA inclusion could regulate the homeostatic inflammatory control baseline at a physiological lower level compared to broilers fed with non-added PFA. Moreover, the effects of dietary PFA addition on the *APO1* and *FOS* genes of the *MAPK* pathway at the broiler jejunum could additionally suggest that PFA inclusion may regulated cellular apoptosis at a “slower” rate. Generally, the findings of this study could be considered in line with evidence from other studies where phytogenic components such as carvacrol, cinnamaldehyde, curcumin, and thymol have been shown to suppress the *NFkB* and *MAPK* signaling pathways in pigs and chickens (Liang et al., 2014; Huang and Lee, 2018).

Furthermore, PFA inclusion resulted in modulation of critical genes related to *mTOR* pathway noted by the reduced expression of the *TSC2* and the increased expression of *4EBP1*, compared to the non-PFA supplemented broilers. As mentioned in the literature *TSC2* is known to inhibit cellular translation by inhibiting the phosphorylation of *4EBP1* (Inoki et al., 2003). Given that the downregulation of *TSC2* protect cells from energy deprivation-induced apoptosis (Inoki et al., 2003; Ma et al., 2005) and the upregulation of *4EBP1* is linked with protein synthesis, it could be postulated that PFA inclusion in this study beneficially affected cellular growth. The latter may in turn explain the benefits evidenced for broiler performance upon PFA supplementation especially in the case of broilers fed the reduced spec diet.

The findings of our previous study (Griela et al., 2021) showed that diet type 95% (ME and CP specs) and the non-PFA inclusion reduced boiler performance and the antioxidant response mediated via the *Nrf2* pathway, compared either to 100% specs diet or the PFA inclusion, respectively (Griela et al., 2021). The findings of the present study explain further the reduced broiler performance seen when broilers were fed low spec diet or diets without PFA. In particular, ample evidence has been provided with respect to the regulation of critical components relevant for inflammation control, apoptosis and nutrient sensing and cell growth. The high spec diet was shown to regulate gut homeostasis response with respect to the above pathways to a “lower” physiological level, suggesting that more energy and nutrients could become available for production purposes. Interestingly PFA inclusion also acted in a similar manner with the high spec diet with respect to the modulation of the pathway components assessed. The latter results provide a mechanistic insight documenting further the capacity of PFA to counteract performance losses at reduced diet specs. Further studies at proteomic and metabolomic levels are expected to generate further knowledge on the topic.
